# Anthropogenic enrichment of the chemical composition of bottom sediments of water bodies in the neighborhood of a non-ferrous metal smelter (Silesian Upland, Southern Poland)

**DOI:** 10.1038/s41598-019-51027-w

**Published:** 2019-10-08

**Authors:** Robert Machowski, Martyna A. Rzetala, Mariusz Rzetala, Maksymilian Solarski

**Affiliations:** University of Silesia in Katowice, Faculty of Earth Sciences, Bedzińska 60, 41-200 Sosnowiec, Poland

**Keywords:** Urban ecology, Hydrology, Hydrology, Natural hazards, Natural hazards

## Abstract

An assessment was carried out of the anthropogenic enrichment of the chemical composition of the bottom sediments of water bodies situated in an area with an urban and industrial character (63.7% of the total area). The endorheic catchments of the water bodies studied are lithologically uniform with sandy formations accounting for more than 90% of the surface area. On the basis of geoaccumulation index values, it was found that the bottom sediments of the water bodies studied were contaminated with the following elements: Cd, Zn, S, As, Pb, Sr, Co, Cr, Cu, Ba, Ni, V, Be, in degrees ranging from moderate to extreme, with lower contamination (or absence of contamination) with the same elements being found in the formations present in the vicinity and in the substrate of the basins of water bodies. It was found that one consequence of the fact that these water bodies are located in urban and industrial areas is that there is anthropogenic enrichment of the chemical composition of bottom sediments with certain basic components (organic matter, Mn, Ca and P compounds) and trace elements: Cd, Zn, Pb, Sb, As, Cu and Co, Br, Ni, S, Be, Cs, Sr, V, Cr, Sc, Ba, U, Ce, Eu and Th, with virtually no enrichment of sediments with the other basic and trace components analysed (La, Rb, K_2_O, Nd, Sm, Na_2_O, Hf, SiO_2_, Zr).

## Introduction

The basins of water bodies serve as sedimentary basins and the sediments within them record the phenomena and processes occurring in the environment^1,2^. Since they constitute polygenetic material, the chemical composition of bottom sediments is largely dependent on natural conditions and human pressure^3–7^. The chemical composition of bottom sediments in water bodies is a consequence of natural processes (e.g. the weathering of rocks, volcanic phenomena, naturally occurring fires) or supply from anthropogenic sources (e.g. settlement, agriculture, industry, transport)^8^. The basic composition of bottom sediments and the concentration of trace elements are good indicators of the pollution of water ecosystems and of the environmental conditions prevailing in the catchment area^9–11^.

The chemical composition of bottom sediments in inland water bodies is usually analysed in terms of human impact, using indicators that take the geochemical background of the sediments into account^12–15^. A relatively new geoecological approach is an attempt to assess the degree of anthropogenic enrichment of the chemical composition of sediments against the background geochemical characteristics of the surface formations representative of the substrate and the area in the vicinity of water bodies^8^. Being able to compare contamination levels for lithologically uniform formations subject to differing degrees of human pressure is tantamount to conducting a field experiment.

The purpose of the study is to compare the anthropogenic enrichment of the chemical composition of bottom sediments with the geochemical characteristics of sediments in the vicinity of the basins of water bodies basins and in their substrate. Such geochemical testing of sediments is particularly important in the case of water bodies that have a variety of economic uses, especially in densely populated areas where there is usually a shortage of water that is conventionally considered clean.

## Materials and Methods

### Research area

**Figure 1:**
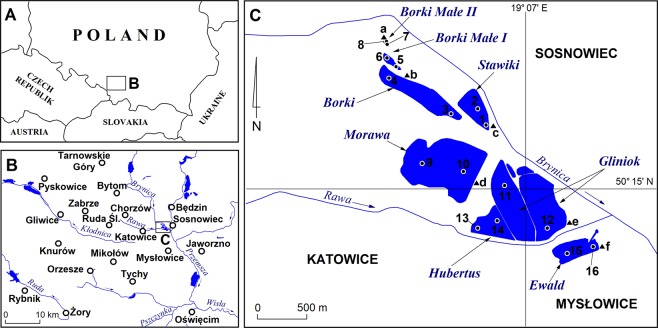
The water bodies are situated around the place where the Rawa River enters the Brynica River with bottom sediment sampling sites marked (samples labelled 1–16) and surface formation sampling sites marked (samples labelled a–f). The samples were collected for geochemical analysis purposes.

Field research covered a group of several water bodies in southern Poland (Fig. 1). These are located in the central part of the Silesian Upland on the boundaries of three cities: Katowice, Sosnowiec and Mysłowice. It is a central part of a heavily industrialised and urbanised region in southern Poland^16^. In this area, the long-term exploitation of mineral resources and the growth of manufacturing industry have resulted in the greatest degree of urban development in Poland^8^. Economic development processes in the region were accompanied by a transformation of the natural environment that exceeded all acceptable limits. The area selected for the study is a perfect example of this since it was subject to urban and industrial impacts for many decades, including the operation of a zinc and lead ore smelting plant that was harmful to the environment and dangerous to human health and life.

The smelting activity in the areas adjacent to the studied reservoirs has been carried out for nearly two centuries^17,18^. From the very beginning, zinc was produced in the smelter on the basis of regional deposits of calamine and sphalerite. Later, in connection with the development of new technologies, the plant also smelted cadmium, as well as lead and silver from galena. In the middle of the 20th century, the production and processing of copper and nickel began. In the technological processes used in the smelter, metal ores underwent significant transformations of phase and chemical composition^19^. As a result of non-ferrous metal smelting, large amounts of slag were produced. It contains not only the residues of elements from the smelting of ores (Zn, Pb and S) but also from technological additives used for ore smelting, e.g. Si, Al, Ca, Mg, K^19^. The waste from non-ferrous metal smelting was used in the area of the smelter for road and industrial construction, construction of railway embankments, as well as in the process of the reclamation of extraction workings^2,8,11^.

The water bodies in question formed in the sand quarries that operated there in the 20^th^ century. A characteristic morphogenetic feature of the water bodies examined is the similarity of their basins in terms of the lithology in their vicinity and that of their substrates^20^. The origins of the water retained in them are also similar. Water in endorheic bodies comes primarily from precipitation, underground seepage and runoff^11^. No hydraulic connections between the waters in the river beds and the waters of the neighbouring water reservoirs were found during the field studies. Watercourses are regulated and protected against infiltration of polluted water into the ground. They were also subject to clogging, carrying large quantities of fine bed load – mainly sewage material. Due to the limited possibilities of supplying the studied water reservoirs, the supply of substances from the troposphere, both in the form of dry deposition and with precipitation, contributes significantly to the quantitative and qualitative formation of their bed sediment covers. The quantities of this supply – estimated since data from the Centre for Environmental Testing and Control (Ośrodek Badań i Kontroli Środowiska) in Katowice – have changed over the years and show significant spatial differences. While in the 1970s dust deposition in the area of the studied water reservoirs was the highest in history, at the level of hundreds or thousands of t/km^2^ per year, since the end of the late 1990s dust deposition has reached the order of tens of t/km^2^ per year^20^. Therefore, in the vicinity of non-ferrous metal smelters, much larger quantities of pollutants were discharged into the studied water reservoirs than in the neighbouring areas (which were not urbanised or industrialised), where dust deposition ranged from several to several dozen t/km^2^ per year.

In their morphometry, these basins resemble the bottoms and boundaries of former mineral workings that were slightly modified in the period when these were prepared for flooding. They are small in surface area, with a maximum depth of just a few metres and limited capacity (Table 1). The Stawiki, Morawa, Hubertus, Gliniok and Borki water bodies are used for recreational purposes. The Ewald water body is not used for any economic purpose owing to the fact that non-ferrous metallurgical waste is stored in direct contact with its basin. The Borki Małe I and II water bodies are not used for any economic purpose.

**Table 1:** Morpho- and hydrometric parameters of the water bodies studied (after Rzetała^20^; revised and supplemented).

Water body name and number of samples (Fig. 1)	Year of creation	Area	Average depth	Total capacity	Electrolytic conductivity	Nitrates (NO_3_^−^)	Phosphates (PO_4_^3−^)
		[ha]	[m]	[dam^3^]	[μS/cm]	[mg/dm^3^]	[mg/dm^3^]
Stawiki (1, 2)	1955	7.6	1.7	131	784.5	26.0	0.09
Morawa (9, 10)	1965	34.7	2.0	693	380.0	37.8	3.49
Hubertus (13, 14)	1928	6.7	2.1	142	1102.0	21.0	0.14
Gliniok (11, 12)	1928	38.7	2.1	824	512.1	2.2	0.06
Ewald (15, 16)	1928	6.7	2.1	140	16500.0	6.6	1.11
Borki (3, 4)	1965	12.0	1.7	202	311.0	2.2	0.09
Borki Małe I (5, 6)	1965	1.1	1.0	11	372.3	12.8	0.05
Borki Małe II (7, 8)	1965	0.9	1.0	9	395.2	15.9	0.16

### Field research

Basic forms of land use were identified and samples of sediments and of material representative of the vicinity (and hence of the substrate) of water body basins were collected during field mapping of the study area (preceded by the analysis of topographic, geological and hydrological maps). The following forms of land use were identified: forests and forest planting together with cultivated green areas, grasslands (including wasteland), arable land, land covered with water, built-up areas, industrial areas including industrial wasteland and communication infrastructure.

Bottom sediment samples were collected using the Beeker sediment core sampler (04.20.S.A. version, manufactured by Eijkelkamp) and a van Veen sampler with a capacity of 1.25 dm^3^ or 2.50 dm^3^. Before samples were placed in polyethylene bags, mixed samples were compiled that were representative of the vertical profile of sediments^21^. Sampling was conducted in accordance with the principle of an even distribution of sedimentary cover samples. Two samples were collected from each water body, accounting for the morphometric variation of the basin in question.

After digging a small pit, samples of sediments that were representative of the vicinity (and thus of the substrate) of the water bodies were collected directly to polyethylene bags from a depth of ca. 20 cm below the pit level, i.e. ca. 50 cm below ground level.

### Laboratory research

Laboratory tests were carried out at the Laboratory for Soil and Rock Analysis of the Faculty of Earth Sciences of the University of Silesia in Sosnowiec (Poland) and at Activation Laboratories Ltd. at Ancaster (Canada)^21^.

The mechanical composition of sediments was determined using the sieving and areometric methods^22^. During the preparation of samples for geochemical tests, the material was ground in an agate mortar and the <0.063 mm fraction was isolated using chemically inert sieves.

Chemical composition was determined using inductively coupled plasma (ICP) atomic emission spectrometry and instrumental neutron activation analysis (INAA) in accordance with the standards applied at Activation Laboratories Ltd^23,24^.

The ICP method was used to determine the concentrations of the following elements: SiO_2_, Al_2_O_3_, Fe_2_O_3_, MnO, MgO, CaO, Na_2_O, K_2_O, TiO_2_, P_2_O_5_, loss on ignition, Ba, Be, Sr, V, Y, Zr^23^. Samples were prepared and analysed in batches with each batch containing a method reagent blank, certified reference material and 17% replicates. The samples analysed were mixed with lithium metaborate and lithium tetraborate and fused in an induction furnace. Subsequently, the molten material was poured into a solution of 5% nitric acid containing an internal standard and mixed continuously for about 30 minutes until it dissolved completely^23,25^. The samples were then tested for the presence of major oxides and of selected trace elements on a combination simultaneous-sequential Thermo Jarrell-Ash ENVIRO II ICP spectrometer^23,26^. Using the same method, the content of Cd, Cu, Pb, Ni, S and Zn was determined after the complete dissolution of 0.25 g aliquots. The sample aliquot was digested with a mixture of HClO_4_, HNO_3_, HCl, and HF at 200 °C to fuming and was then diluted with aqua regia^23^.

During the ICP analysis, reagent blanks with and without the lithium borate flux were analysed alongside the method reagent blank. Interference correction verification standards were analysed as well^10,23^. Multiple USGS and CANMET certified reference materials were used for calibration (two standards for every group of ten samples) in order to bracket groups of samples. Internal standards were also added to the sample solution, which was then further diluted. USGS and CANMET certified reference materials were used for calibration purposes. A proprietary methodology was used to introduce the sample into the Perkin Elmer SCIEX ELAN 6000 mass spectrometer^23^. The precision and accuracy of the analyses conducted are as follows: a) at the lower detection limit: +/− 100%; b) at 10 times the lower detection limit: +/− 15–25%; c) at 100 times the lower detection limit: better than 10%^23^.

The INAA method was used to determine the concentrations of the following elements: As, Br, Ce, Co, Cr, Cs, Eu, Hf, La, Nd, Rb, Sc, Sb, Sm, Th and U^21,23^. A 1 g aliquot was placed in a polyethylene vial and irradiated with flux wires and an internal standard (one for 11 samples) at a thermal neutron flux of 7 × 10^12^ n cm^−2^ s ^−1^. After a delay of seven days introduced to allow Na-24 to decay, the samples were counted using a high purity Ge detector with a resolution higher than 1.7 KeV for the 1332 KeV Co-60 photopeak^23,27^. The decay-corrected activities were compared to a calibration obtained with the use of multiple certified international reference materials using flux wires. From 10% to 30% of the samples were rechecked by running the measurement again. The standard present served only to check measurement accuracy and was not used for calibration purposes^23^. The precision and accuracy of the analyses conducted are as follows: a) at the lower detection limit: +/− 100%; b) at 10 times the lower detection limit: +/− 10–15%; c) at 100 times the lower detection limit: better than 5%^23^.

Lower detection limits were as follows: for SiO_2_, Al_2_O_3_, Fe_2_O_3_, MnO, MgO, CaO, Na_2_O, K_2_O, P_2_O_5_–0.01%; for TiO_2_–0.005%, and for S – 0.001%. The lower quantification limit for trace elements was as follows: 5.0 ppm (Nd, Pb, V), 3.0 ppm (Ba, Ce), 2.0 ppm (As, Rb, Sr, Zr), 1.0 ppm (Be, Br, Co, Cr, Cu, Ni, Y, Zn), 0.5 ppm (Cd, Cs, Hf, Th, U), 0.2 ppm (La, Sb) and 0.1 ppm (Eu, Sc, Sm)^23^.

### Statistics

In order to assess the contamination of bottom sediments in the water bodies studied, the geoaccumulation index (Eq. 1) developed by Müller was used^8,12^. Values of the geoaccumulation index make it possible to determine seven classes of sediment quality: I_geo_ ≤ 0.0 – practically uncontaminated sediments; 0.0 < I_geo_ < 1.0) – uncontaminated to moderately contaminated sediments; 1.0 < I_geo_ < 2.0 – moderately contaminated sediments; 2.0 < I_geo_ < 3.0 – moderately to heavily contaminated sediments; 3.0 < I_geo_ < 4.0 – heavily contaminated sediments; 4.0 < I_geo_ < 5.0 – heavily to extremely contaminated sediments; I_geo_ > 5.0 – extremely contaminated sediments^12,20,21^.

1 $${I}_{geo}={\log }_{2}\frac{{C}_{n}}{1.5{B}_{n}}$$

where:

I_geo_ – geoaccumulation index;

C_n_ – the concentration of the element in question in bottom sediments;

B_n_ – geochemical background for the metal in question;

1.5 – coefficient expressing the natural variation in the content of the metal in question in the environment.

In the calculation of the geoaccumulation index, the regional geochemical background values established by Lis and Pasieczna^28^ have been used. For individual elements this background was determined at: 6.0 mg/kg – As, 98 mg/kg – Ba, 0.6 – Be, 2.5 mg/kg – Cd, 4.0 mg/kg – Co, 9.0 mg/kg – Cr, 15.0 mg/kg – Cu, 11.0 mg/kg – Ni, 59.0 mg/kg – Pb, 0.052% – S, 24.0 mg/kg – Sr, 12.0 mg/kg – V and 259.0 mg/kg – Zn. The regional geochemical background values for individual elements are conditioned by the peculiar geological structure of Upper Silesia and the presence of many mineral resources in the region^17,18,20,28–30^.

The I_AP_ index was used to assess the anthropogenic enrichment of deposits with toxic metals, non-metals and metalloids (Eq. 2). The anthropogenic enrichment factor of bottom sediments indirectly reflects the effectiveness of accumulation of allochthonous and autochthonous matter in the water body; this accumulation is often identified with contamination. For a given element, the I_AP_ value is the ratio of its mean content in bottom sediments and its mean content in the sediments in the vicinity of the basin and its substrate (the mean value can be regarded as the result of tests of so-called mixed samples or as the mean value for multiple samples tested). An I_AP_ value greater than unity (I_AP_ > 1.0) indicates that the bottom sediments have been enriched. This value increases as the ratio of the concentration of the element in question in bottom sediments to its concentration in the vicinity of the basin increases. The index value drops below 1.0 (0.0 < I_AP_ > 1.0) if the concentration of the element in question in the sediments is lower than its concentration in the formations surrounding the basin; this indicates that there has been no enrichment of bottom sediments. This index is calculated as follows^11,31^:

2 $${I}_{AP}=\frac{{C}_{BS}}{{C}_{SR}}$$

where:

I_AP_ – the anthropogenic enrichment factor for bottom sediments (dimensionless number);

C_BS_ – the average concentration of the substance in question in bottom sediments of the water body;

C_SR_ – the average concentration of the element in question in substrate sediments and in the vicinity of the basin.

## Results

### Lithological properties and land use

**Figure 2:**
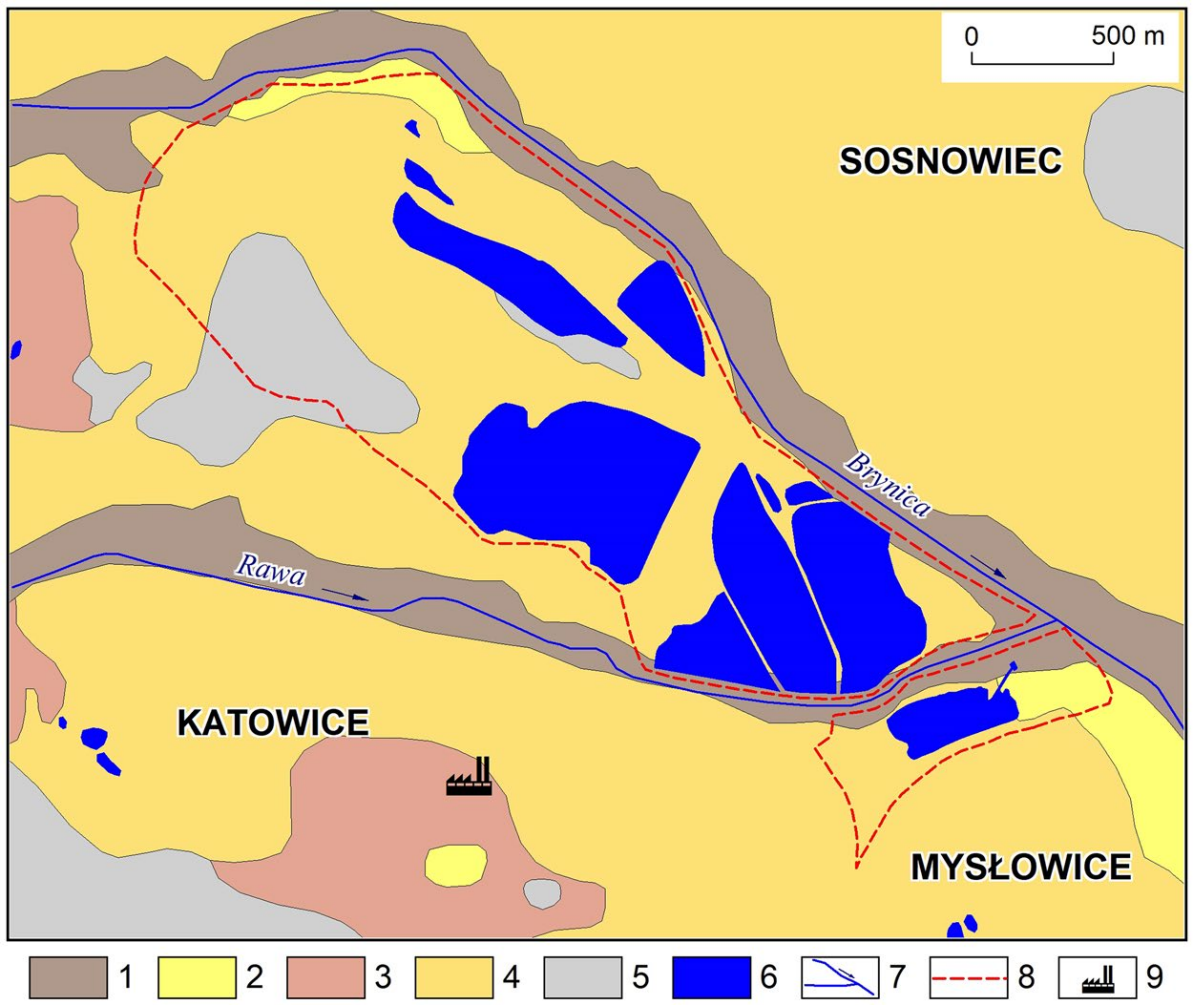
Surface formations in the catchments of endorheic water bodies and in their surroundings (Detailed…^61^ simplified and supplemented): 1 – fluvial deposits in general (Holocene); 2 – sands and gravels on accumulation terraces (Pleistocene); 3 – sandy and silty boulder clay eluvia (Pleistocene); 4 – glacial and fluvioglacial sands and gravels (Pleistocene); 5 – grey shales with sandstones and coal, sandstones, conglomerates, coal (Carboniferous); 6 – water bodies; 7 – surface watercourses; 8 – catchment boundaries; 9 – non-ferrous metal smelter.

The study area has a peculiar geological structure which enabled a unique geochemical comparison to be made between the sediments in the vicinity and the substrates of the basins of the water bodies and of the bottom sediments. A cover of Pleistocene sediments rests on a geological foundation formed by Carboniferous rocks, and the river valleys are filled with Holocene sediments. The most widespread of the surface formations are Pleistocene sediments (79.7% of total area). These are sands and gravels of glacial and fluvio-glacial origin alongside Pleistocene sandy and silty boulder clay eluvia and sands and gravels on accumulation terraces. In lithological terms, these are mainly sandy and gravelly deposits, which attracted economic interest and started to be exploited in the first half of the 20^th^ century. Holocene fluvial deposits were found in 12.6% of the area. In ca. 7.7% of the area, Carboniferous grey shales with sandstones and coal, sandstones and conglomerates are exposed (Fig. 2).

**Figure 3:**
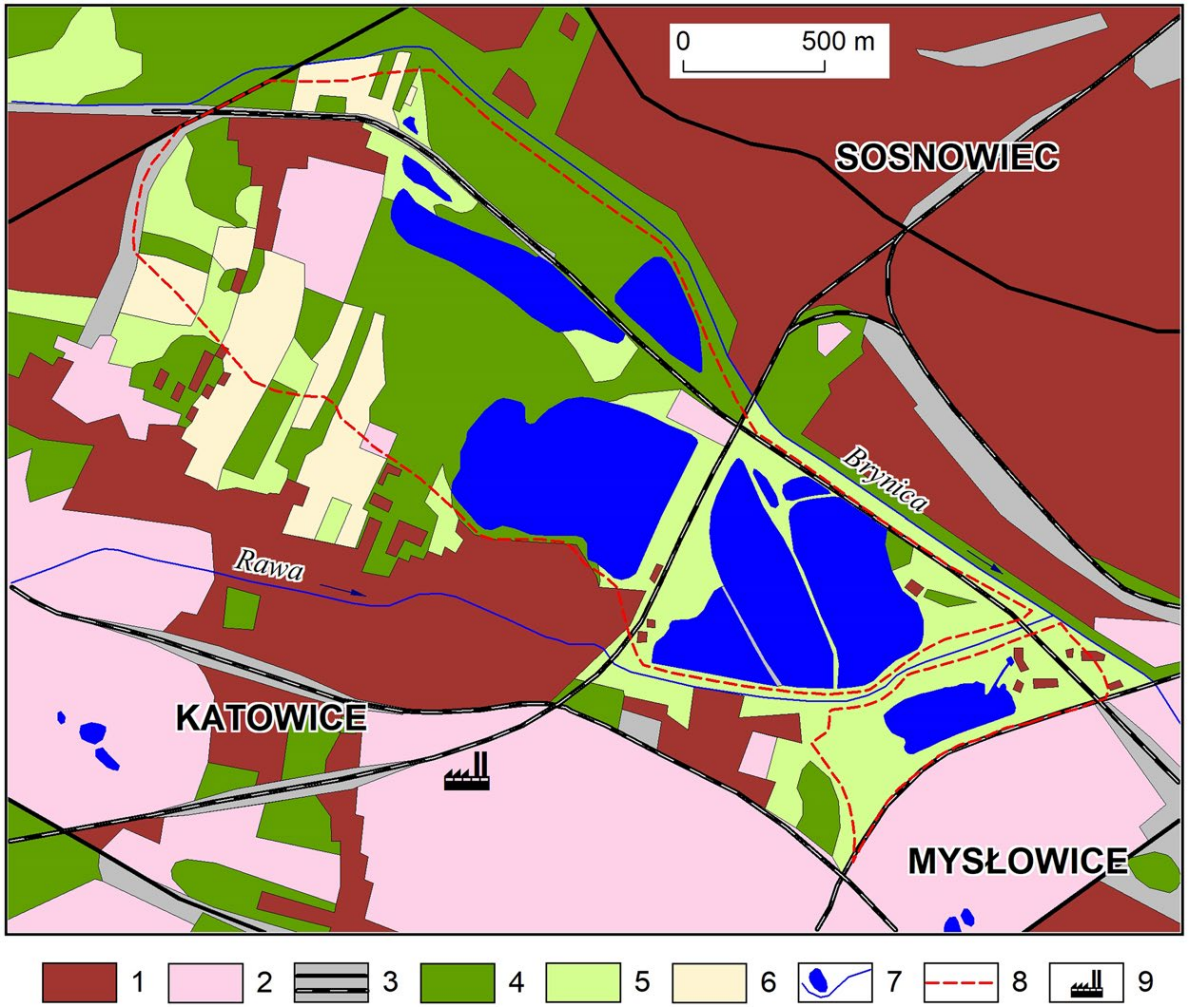
The main forms of land use in the catchments of endorheic water bodies and in their vicinity: 1 – built-up urban areas; 2 – industrial areas (including industrial wasteland); 3 – communications infrastructure; 4 – forests, plantations, cultivated green areas, 5 – meadows (including abandoned agricultural land); 6 – agricultural land; 7 – watercourses and water bodies; 8 – boundaries of endorheic water body catchments; 9 – non-ferrous metal smelter.

The water bodies are surrounded by typical urban and industrial areas. Built-up urban areas are the most prevalent (34.4%) within ca. 14 km^2^ (Fig. 3); industrial areas together with industrial wasteland account for 21.2% and 8.1% is occupied by communications infrastructure and associated unused land. Forests, plantations, scrub and cultivated green areas occupy 16.3% of the surface area, and meadows and arable land account for 8.9% and 2.9% respectively. The surface covered with water is more than 1.1 km^2^ (8.2%).

### The chemical composition of the sediments

In geochemical terms, the sediments studied are clearly differentiated. This concerns both their basic composition and their content of trace elements (Tables 2 and 3).

**Table 2:** Basic composition of bottom sediments in endorheic water bodies.

Component	Unit	Lower detection limit	Minimum	Median	Maximum	Arithmetic means	Standard deviation
SiO_2_	%	0.01	21.75	51.20	80.76	49.76	16.42
Al_2_O_3_	%	0.01	6.71	8.56	11.70	9.06	1.55
Fe_2_O_3_	%	0.01	3.06	6.32	13.23	7.59	3.34
MnO	%	0.01	0.05	0.34	1.02	0.36	0.24
MgO	%	0.01	0.35	0.94	1.94	1.03	0.37
CaO	%	0.01	0.62	2.13	28.24	6.38	8.47
Na_2_O	%	0.01	0.34	0.51	0.94	0.57	0.18
K_2_O	%	0.01	0.87	1.57	2.10	1.49	0.31
TiO_2_	%	0.005	0.36	0.69	0.83	0.64	0.16
P_2_O_5_	%	0.01	0.07	0.19	0.30	0.16	0.07
LOI	%	0.01	4.20	18.11	32.19	18.55	7.99

**Table 3:** Trace elements in the bottom sediments of endorheic water bodies.

Component	Unit	Minimum	Median	Maximum	Arithmetic means	Standard deviation
As	mg/kg	14.0	90.0	330.0	110.2	96.7
Ba	mg/kg	430.0	545.5	1940.0	712.0	441.2
Be	mg/kg	1.0	3.0	7.0	3.6	1.5
Br	mg/kg	2.0	9.5	34.0	11.1	9.5
Cd	mg/kg	8.5	54.6	444.0	113.1	123.8
Ce	mg/kg	56.0	98.0	113.0	91.6	14.9
Co	mg/kg	10.0	33.0	90.0	34.1	17.6
Cr	mg/kg	69.0	135.0	203.0	134.2	36.8
Cs	mg/kg	3.8	7.2	14.9	7.7	2.7
Cu	mg/kg	20.0	117.0	298.0	151.1	95.1
Eu	mg/kg	1.0	2.0	2.6	1.8	0.4
Hf	mg/kg	2.9	15.1	36.0	17.3	10.8
La	mg/kg	25.0	44.8	52.2	44.0	7.5
Nd	mg/kg	5.0	35.0	49.9	33.0	10.9
Ni	mg/kg	20.0	57.0	148.0	66.0	34.5
Pb	mg/kg	130.0	2355.0	3200.0	2040.8	1064.6
Rb	mg/kg	20.0	47.5	90.0	50.3	19.2
S	%	0.3	2.0	4.7	2.4	1.3
Sb	mg/kg	3.5	37.1	80.6	33.9	20.6
Sc	mg/kg	6.9	11.1	16.3	11.2	2.2
Sm	mg/kg	4.2	7.8	9.2	7.4	1.3
Sr	mg/kg	89.0	153.5	1107.0	266.3	306.8
Th	mg/kg	6,0	10,9	24,0	11,3	4,6
U	mg/kg	1.3	5.3	7.9	4.9	1.6
V	mg/kg	44.0	81.0	140.0	84.8	22.0
Y	mg/kg	14.0	30.5	38.0	29.2	6.1
Zn	mg/kg	805.0	4895.0	38400.0	12724.7	13274.6
Zr	mg/kg	72.0	458.0	1195.0	543.5	381.5

The geochemistry of the sediments present in the vicinity of water bodies is equally varied. As concerns their basic composition, the following substances were identified: SiO_2_ (79.90–86.10%), Al_2_O_3_ (5.75–9.20%), Fe_2_O_3_ (1.06–2.09%), MnO (0.02–0.04%), MgO (0.21–0.38%), CaO (1.16–1.85%), Na_2_O (0.60–0.92%), K_2_O (1.66–1.94%), TiO_2_ (0.62–0.78%), P_2_O_5_ (0.04–0.08%). Loss on ignition ranges from 2.10% to 5.2% depending on the sample.

The contents of trace elements in the sediments identified as belonging to the substrate of the basins of water bodies (Fig. 1 – samples a–f), i.e. present in the vicinity of the water bodies studied, were as follows: 5.0–7.0 mg/kg – As, 474.0–530.0 mg/kg – Ba, 1.0–1.0 mg/kg – Be, 2.0–2.0 mg/kg – Br, 0.5–0.5 mg/kg – Cd, 76.0–86.0 mg/kg – Ce, 2.0–6.0 mg/kg – Co, 53.0–72.0 mg/kg – Cr, 1.7–2.8 mg/kg – Cs, 8.0–20.0 mg/kg – Cu, 1.4–2.1 mg/kg – Eu, 20.6–35.8 mg/kg – Hf, 48.7–51.0 mg/kg – La, 34.0–46.0 mg/kg – Nd, 10.0–16.0 mg/kg – Ni, 19.0–33.0 mg/kg – Pb, 50.0–80.0 mg/kg – Rb, 0.38–0.84% – S, 0.6–1.0 mg/kg – Sb, 5.5–8.1 mg/kg – Sc, 7.9–12.0 mg/kg – Sm, 79.0–99.0 mg/kg – Sr, 2.9–5.0 mg/kg – U, 35.0–44.0 mg/kg – V, 34.0–47.0 mg/kg – Y, 75.0–116.0 mg/kg – Zn, 1,050.0–1,195.0 mg/kg – Zr.

## Discussion

### The geochemical properties of the sediments

Variations in the chemical composition of sediments between individual endorheic water bodies are conditioned by the geological substrate, the manner in which the catchment is utilised and the type of atmospheric deposition^9^. The chemical composition of bottom sediments is varied and indicates strong human impact (Tables 2 and 3).

As concerns overall composition, SiO_2_ prevails in almost all samples and the accompanying loss on ignition (measure of organic matter content) is usually inversely proportional to SiO_2_ content. SiO_2_ content ranges from 21.75% to 80.76% and loss on ignition ranges from 4.20% to 32.19%. Generally, the correlation between higher SiO_2_ content and lower loss on ignition results from catchment conditions (substrates consisting of sandy Pleistocene formations) and human impact (former mineral workings without any overburden and deposit). A high loss on ignition indicates a significant organic matter content in the sediments^10^. The coastal zones of lakes, which are often colonised by compact stands of rushes, play a major role in determining the amount of this matter^32^. These stands exhibit high bioproductivity and the plant fall originating there as autochthonous matter has a significant impact on the chemical composition of sediments^8,9^.

Apart from organic matter and silica, bottom sediments also include the following minerals or their components as their basic building materials: Al_2_O_3_ and Fe_2_O_3_ as well as manganese, magnesium, calcium, sodium, potassium, titanium and phosphorus compounds. Their percentage shares in sediments are also dependent on catchment lithology and the nature of the human impact^20^. Against the background of all test results concerning basic components, Al_2_O_3_ content (in the range from 6.71% to 11.70%) stands out, which may indicate a relationship between the concentration of this substance in the sediments and the long-standing activity of a nearby non-ferrous metal smelter. Fe_2_O_3_ concentration in the bottom sediments examined (from 3.06% to 12.23%) should be considered typical of water bodies in sand workings situated in catchments with a high proportion of post-glacial sandy formations^15^. In some places, Fe_2_O_3_ may arise as a consequence of the presence of traces of bog iron ores. In addition the impact of local landfill sites or metallurgical industrial processes on bottom sediments cannot be ruled out. The high content of CaO (in the 0.62%–28.24% range) is related to the waste from non-ferrous smelters present in water body sediments, although the high share of calcium in the sediments in neighbouring areas is due to the presence of carbonate Triassic formations in the geological structure and is related to human agricultural activity. The fairly universal and uniform presence of phosphorus in bottom sediments may be attributed to natural processes (e.g. the leaching of bioelements from the rocks present within the catchment) as well as to anthropogenic sources (e.g. discharges of household, municipal and industrial sewage and run-off from agricultural land)^20^.

In addition to the macroelements, the chemical composition of bottom sediments also includes a number of trace elements. Their presence in the environment is determined both by natural processes (e.g. the weathering of rocks) and by their supply from anthropogenic sources (e.g. industrial processes, traffic)^8^. Of special geoecological significance are the quantitative and qualitative differences in the presence of some metals (especially of so-called heavy metals), non-metals and metalloids. Among the elements identified in bottom sediments, Zn and Pb were present in the highest average amounts (thousands of mg/kg). Ba, Zr, Sr, Cu, Cr, Cd and As are present in average concentrations of hundreds of mg/kg. Average concentrations ranging from around a dozen to several dozen mg/kg are reached by Ce, V, Ni, Rb, La, Co, Sb, Nd, Y, Hf, Th, Sc, and Br; the remaining elements are present in smaller amounts (Cs, Sm, U, Be, Eu); the average sulphur content found was 2.4%. The quantities of individual trace elements found in the bottom sediments of the water bodies studied are extremely high on a global scale^20^. This thesis is confirmed by the results of many years of research conducted in different parts of the world where much lower concentrations of the elements identified were found^33–51^. Record-high concentrations of the measured elements – indicated in the analysis of the results of calculations of standard deviation for the raw data (Table 3) and the anthropogenic enrichment factor of bed sediments (Table 4) – confirm the references to the geochemical background of the formations occurring in the upper part of the Earth’s crust^52–54^, as well as water sediments found in Europe^55^. In addition to the extremely high concentrations of individual elements in the bottom sediments studied, they also exhibit a quite large spatial variability (Table 3).

**Table 4:** Statistical characteristics of the anthropogenic enrichment index of bottom sediments.

Component	Minimums	Median	Maximum	Arithmetic means	Standard deviation
SiO_2_	0.26	0.62	0.97	0.60	0.20
Al_2_O_3_	1.01	1.29	1.76	1.36	0.23
Fe_2_O_3_	1.67	3.45	7.23	4.15	1.83
MnO	1.67	11.33	34.00	11.94	7.88
MgO	1.25	3.36	6.93	3.66	1.31
CaO	0.44	1.50	19.89	4.49	5.97
Na_2_O	0.43	0.65	1.19	0.72	0.22
K_2_O	0.46	0.84	1.12	0.79	0.16
TiO_2_	0.49	0.96	1.15	0.89	0.23
P_2_O_5_	1.17	3.08	5.00	2.74	1.17
LOI	1.15	4.98	8.84	5.10	2.19
As	2.33	15.00	55.00	18.36	16.11
Ba	0.85	1.08	3.84	1.41	0.87
Be	1.00	3.00	7.00	3.56	1.55
Br	1.00	4.75	17.00	5.53	4.73
Cd	17.00	109.10	888.00	226.24	247.52
Ce	0.69	1.21	1.40	1.13	0.18
Co	2.50	8.25	22.50	8.52	4.39
Cr	1.08	2.11	3.17	2.10	0.57
Cs	1.65	3.11	6.48	3.33	1.17
Cu	1.82	10.64	27.09	13.73	8.65
Eu	0.59	1.18	1.53	1.07	0.24
Hf	0.11	0.56	1.35	0.65	0.41
La	0.50	0.89	1.04	0.88	0.15
Nd	0.12	0.83	1.19	0.79	0.26
Ni	1.67	4.75	12.33	5.50	2.87
Pb	4.81	87.22	118.52	75.59	39.43
Rb	0.33	0.79	1.50	0.84	0.32
S	0.51	3.69	8.64	4.35	2.38
Sb	4.38	46.38	100.75	42.31	25.74
Sc	1.03	1.65	2.43	1.67	0.33
Sm	0.44	0.82	0.97	0.78	0.13
Sr	1.02	1.76	12.72	3.06	3.53
Th	0.55	0.99	2.18	1.02	0.42
U	0.37	1.51	2.26	1.39	0.45
V	1.13	2.08	3.59	2.17	0.56
Y	0.33	0.73	0.90	0.69	0.14
Zn	7.74	47.07	369.23	122.35	127.64
Zr	0.06	0.40	1.05	0.48	0.34

The very high concentrations of zinc and lead in water body sediments are typical of areas situated in close proximity to ore smelting centres and waste dumps from non-ferrous smelters. Owing to the proximity of a non-ferrous smelting plant (1.0–2.0 km) to the water bodies studied, the area is contaminated with these elements. The concentrations of zinc (805.0–38,400.0 mg/kg) and lead (130.0–3,200.0 mg/kg) in bottom sediments exceed many times the average contents of these elements in the Earth’s crust (71.0–83.0 mg/kg for Zn, 4.0–20.0 mg/kg for Pb) as stated by Taylor and McLennan^54^. The concentrations of these elements are also much higher than the regional geochemical background for water sediments determined by Lis and Pasieczna^28^ at 259.0 mg/kg for zinc and 59.0 mg/kg for lead.

In a similar manner to zinc and lead, the concentrations of other heavy metals (Cu, Cr, Cd, Ni) found in the bottom sediments of water bodies situated within the zones affected by non-ferrous smelting plants are very high^16^. The regional geochemical background for copper in water body sediments corresponds to the average amount of this element present in the Earth’s crust at 15 mg/kg^28,56^, while in the sediments studied its concentration ranges from 20.0 to 298.0 mg/kg. The concentrations of chromium (69.0–203.0 mg/kg), cadmium (8.5–444.0 mg/kg) and nickel (20.0–148.0 mg/kg) found in sediments are significantly higher than the natural concentrations of these elements in the region determined by Lis and Pasieczna^28^, which amount to 9.0 mg/kg (Cr), 2.5 mg/kg (Cd) and 11.0 mg/kg (Ni). The geochemical background for chromium in various sedimentary rocks ranges from 5 to 120 mg/kg^56^. The average cadmium content in the Earth’s crust is 0.1 mg/kg and nickel content in the Earth’s crust averages 20–135 mg/kg^54^.

The presence of cobalt in aquatic ecosystems may be caused to a large extent by the denudation of the natural rock and soil environment^51^. Cobalt, which is present in the lithosphere at ca. 40 mg/kg^56^, was detected in the bottom sediments of the water bodies examined in amounts ranging from 10.0 to 90.0 mg/kg. In each case the concentrations of Co are higher than the regional geochemical background determined by Lis and Pasieczna^28^ at 4.0 mg/kg.

Some of the trace elements found in the sediments are alkaline Earth metals (e.g. beryllium, barium, strontium) and their content in the Earth’s crust is lower than that of calcium and magnesium, which are the most common among this group of elements^56^. Strontium, which is widely used in industry, is present in the bottom sediments of the water bodies studied in amounts ranging from 89.0 mg/kg to 1,107.0 mg/kg. Barium concentrations range from 430.0 mg/kg to 1,940.0 mg/kg. The natural strontium content in crustal rocks is estimated at 350 mg/kg and that of barium at 570 mg/kg^57^.

Beryllium was found in bottom sediments of water bodies in amounts ranging from 1.0 to 7.0 mg/kg, which in most cases corresponds to its natural content (1.0–3.0 mg/kg) in the Earth’s crust^54^, and is greater than or equal to the value (2 mg/kg) typical of sedimentary rocks as determined by Kabata-Pendias and Pendias^56^. The main anthropogenic source of this metal in the environment is the process of fuel combustion and therefore its migration to groundwater is highly influenced by the contact of water bodies with piles of waste rock, dust from power plants and municipal and industrial sewage.

Several of the elements identified belong to so-called lanthanides (cerium, europium, neodymium, samarium) that are present in the Earth’s crust in trace amounts^56^. Although some of them are radiotoxic in higher concentrations, the amounts found in the bottom sediments studied do not pose any environmental threat.

Hafnium and zirconium, which occur together, are so-called transition metals, similar to lanthanum. In the Earth’s outer crust, zirconium is present at concentrations of 167.0 mg/kg on average^56^, and in the sediments studied its concentration ranges from 72.0–1,195.0 mg/kg. Lanthanum, which occurs in the Earth’s crust at 11–30 mg/kg^54^, reaches concentrations from 25.0 mg/kg to 52.2 mg/kg in the sediments studied. Vanadium is a metal used in industry whose natural concentration in the Earth’s crust is estimated at 140 mg/kg; its concentration in the bottom sediments studied is lower (44.0–140.0 mg/kg). Another metal is scandium with a natural concentration in the Earth’s crust of 11 mg/kg^56^; in the sediments studied the level is similar (6.9–16.3 mg/kg). Yttrium is another metal that occurs in the Earth’s crust at a level of 20 mg/kg^54^; in the sediments studied, it was found in similar amounts (14.0–38.0 mg/kg).

Two actinides – thorium and uranium – are present in the samples of bottom sediments examined in amounts (6.0–24.0 mg/kg – Th, 1.3–7.9 mg/kg – U) equivalent to or slightly higher than their natural concentrations in the Earth’s crust, which are assessed by Kabata-Pendias and Pendias^56^ at ca. 12.0 mg/kg and ca. 2.5 mg/kg respectively.

As alkali metals, caesium and rubidium are considered – in spite of being easily soluble – not highly mobile and rapidly adsorbed by clay minerals^56^. In the samples of bottom sediments tested, caesium was found in amounts of 3.8–14.9 mg/kg. Rubidium is present in bottom sediments in amounts ranging from 20.0 mg/kg to 90.0 mg/kg.

Some of the elements identified (arsenic and antimony) are metalloids with properties that are intermediate between metals and non-metals^58^. Their presence in the bottom sediments of the water bodies studied is probably the result of non-ferrous ore mining and metallurgy as well as combustion processes and their fairly widespread use. Arsenic and antimony, whose natural contents in the lithosphere are up to 18 mg/kg and 0.2 mg/kg respectively^56^, were found in amounts ranging from 14.0 to 330.0 mg/kg (arsenic) and from 3.5 to 80.6 mg/kg (antimony).

Bromine is a non-metal which is present in the Earth’s crust at a natural level of ca. 1 mg/kg^56^ while the samples analysed contained 2.0–34.0 mg/kg.

Sulphur content ranges from 0.3% to 4.7%; it is among the common ingredients of bottom sediments in the water bodies analysed which are subject to the impact of human industrial activity.

**Figure 4:**
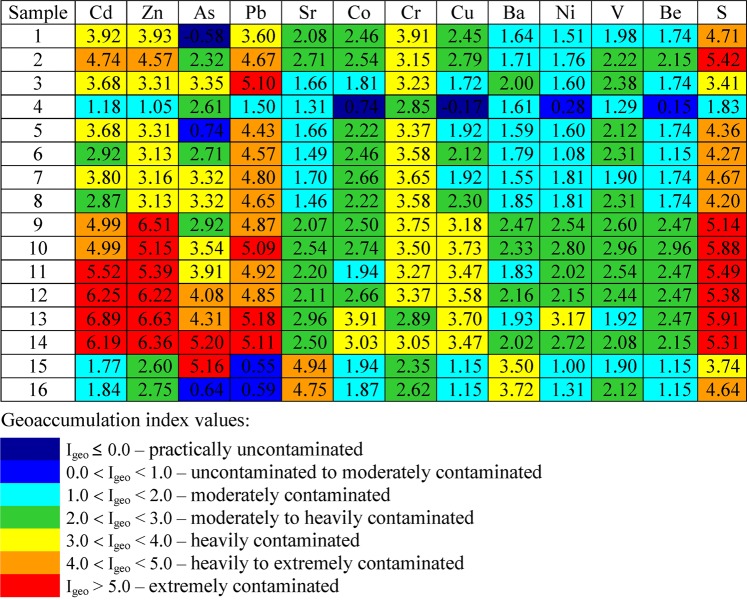
Geoaccumulation index values^12^ for bottom sediments in water bodies.

The bottom sediments in the water bodies studied are characterised by a degree of pollution ranging from moderate to extreme, and for individual elements pollution is completely absent in a few samples only. This is confirmed by geoaccumulation index values (Fig. 4). The highest values of this index include Cd (1.18–6.89), Zn (1.05–6.63), S (1.83–5.91), As (0.64–5.20), Pb (0.55–5.18) and Sr (1.31–4.94). Slightly lower values, although indicative of contamination as well, were found in the case of Co (0.74–3.91), Cr (2.35–3.91), Cu (−0.17–3.73), Ba (1.55–3.72), Ni (0.28–3.17), V (1.29–2.96) and Be (0.15–2.96).

Geoaccumulation index values calculated for bottom sediment samples are in clear contrast with the corresponding figures for the sediment samples representative of the substrate and areas in the vicinity of the basins of water bodies. The calculated I_geo_ ratios based on the average concentrations of the elements analysed indicate:

- no contamination with Cd (I_geo_ of −2.91), Zn (I_geo_ of −1.90), Pb (I_geo_ of −1.71), Cu (I_geo_ of −1.03), As (I_geo_ of −0.58), Co (I_geo_ of −0.58) and Ni (I_geo_ of −0.46);
- the presence of Be (I_geo_ of 0.15) corresponding to an intermediate level between uncontaminated and moderately contaminated sediments;
- moderate contamination of sediments with Ba (I_geo_ of 1.78), Sr (I_geo_ of 1.27) and V (I_geo_ of 1.12);
- the presence of S (I_geo_ of 2.80) and Cr (I_geo_ of 2.25) resulting in a degree of contamination ranging from moderate to heavy.

### Anthropogenic enrichment of sediments

The studies conducted have revealed differences in the basic composition and contents of trace elements between the bottom sediments of water bodies and the formations present in the vicinity (substrate) of these water body basins. In bottom sediments, certain oxides and most trace elements accumulate in quantities that exceed many times the original levels for the area in question (i.e. those before the water bodies formed). The measure of the difference in substance concentrations in the bottom sediments of water bodies as compared to sediments in the substrate (vicinity) of the basins of water bodies is the anthropogenic enrichment index (I_AP_)^8,11^. The index value drops below 1.0 (I_AP_ < 1.0) if the concentration of the element in question in the sediments is lower than its concentration in the formations surrounding the basin. An I_AP_ value greater than unity (I_AP_ > 1.0) indicates anthropogenic enrichment of bottom sediments and increases together with the ratio of the concentration of the substance in question in bottom sediments to its concentration in the vicinity of the basin. It should be understood as an increase in the concentration of the substance in question in bottom sediments against the background of the original chemical composition of the substrate and areas in the vicinity of the basin^8,11^.

In the sediments studied the mean value of the anthropogenic enrichment index for bottom sediments ranges from 0.48 for Zr to 226.24 for Cd with a much greater range for extreme values (Table 4). During the lifetime of the water bodies, the basic composition of their bottom sediments was enriched with organic matter (1.15 < I_AP_ < 8.84) and manganese (1.67 < I_AP_ < 34.00), calcium (0.44 < I_AP_ < 19.89) and phosphorus (1.17 < I_AP_ < 5.00) compounds. Among the trace elements, the highest sediment enrichment has been found with respect to Cd (17.00 < I_AP_ < 888.00), Zn (7.74 < I_AP_ < 369.23), Pb (4.81 < I_AP_ < 118.52), Sb (4.38 < I_AP_ < 100.75), As (2.33 < I_AP_ < 55.00), Cu (1.82 < I_AP_ < 27.09) and Co (2.50 < I_AP_ < 22.50). Enrichment factors below ten were found with respect to: Br (1.00 < I_AP_ < 17.00), Ni (1.67 < I_AP_ < 12.33), S (0.51 < I_AP_ < 8.64), Be (1.00 < I_AP_ < 7.00), Cs (1.65 < I_AP_ < 6.48), Sr (1.02 < I_AP_ < 12.72), V (1.13 < I_AP_ < 3.59) and Cr (1.08 < I_AP_ < 3.17). On average, the enrichment factor was lower still for: Sc (1.03 < I_AP_ < 2.43), Ba (0.85 < I_AP_ < 3.84), U (0.37 < I_AP_ < 2.26), Ce (0.69 < I_AP_ < 1.40), Eu (0.59 < I_AP_ < 1.53) and Th (0.55 < I_AP_ < 2.18). Virtually no enrichment of sediments was found with respect to the other basic components and trace elements analysed (La, Rb, K_2_O, Nd, Sm, Na_2_O, Hf, SiO_2_, Zr).

The values of the anthropogenic enrichment index of bottom sediments in water bodies indicate that basins in which water is retained fulfil the function of local sedimentary basins in which autochthonous as well as transit (allochthonous) pollutants accumulate^11^. The considerable increase in the concentration of some main components and trace elements in bottom sediments against the background of their concentrations in the vicinity of the basin indicates the presence of an important issue with both environmental and social implications. Basins of such water bodies which, like their endorheic catchments, have been transformed by human activity, are in contact with the waste left after processing zinc and lead ores. The long-standing impact of non-ferrous metallurgy amplifies human-induced environmental changes, which are typical of urban and industrial areas.

The anthropogenic enrichment of bottom sediments in the water bodies analysed has the characteristics of contamination. Its equivalent is the presence of the so-called zinc desert in the vicinity of the water bodies^59^. Contamination with heavy metals (and particularly toxic metals) probably explains the high mortality of tench in the Hubertus water body and the disappearance of eels from the Morawa Lake^8,59^. Kostecki^59,60^ states that the heavy metal concentrations recorded in aquatic ecosystems already pose a threat to human health and the concentrations recorded in phyto- and zooplankton, vascular plants and ichthyofauna point to contamination^9^.

## Conclusions

The study area is an urban and industrial one (63.7% of the area in total). The endorheic catchments of the water bodies studied are lithologically uniform with sandy formations accounting for more than 90% of the surface area.

On the basis of geoaccumulation index values it was found that the bottom sediments of the water bodies studied were contaminated with the following elements: Cd, Zn, S, As, Pb, Sr, Co, Cr, Cu, Ba, Ni, V, Be, in degrees ranging from moderate to extreme, with lower contamination (or absence of contamination) with the same elements being found in the formations present in the vicinity and in the substrate of the basins of water bodies.

The consequence of the location of the water bodies studied in urban and industrial areas is the anthropogenic enrichment of the chemical composition of bottom sediments with certain basic components (organic matter, Mn, Ca and P compounds) and trace elements: Cd (17.00 < I_AP_ < 888.00), Zn (7.74 < I_AP_ < 369.23), Pb (4.81 < I_AP_ < 118.52), Sb (4.38 < I_AP_ < 100.75), As (2.33 < I_AP_ < 55.00), Cu (1.82 < I_AP_ < 27.09), Co (2.50 < I_AP_ < 22.50), Br (1.00 < I_AP_ < 17.00), Ni (1.67 < I_AP_ < 12.33), S (0.51 < I_AP_ < 8.64), Be (1.00 < I_AP_ < 7.00), Cs (1.65 < I_AP_ < 6.48), Sr (1.02 < I_AP_ < 12.72), V (1.13 < I_AP_ < 3.59) and Cr (1.08 < I_AP_ < 3.17), Sc (1.03 < I_AP_ < 2.43), Ba (0.85 < I_AP_ < 3.84), U (0.37 < I_AP_ < 2.26), Ce (0.69 < I_AP_ < 1.40), Eu (0.59 < I_AP_ < 1.53) and Th (0.55 < I_AP_ < 2.18). There is virtually no sediment enrichment with the remaining basic and trace components analysed (La, Rb, K_2_O, Nd, Sm, Na_2_O, Hf, SiO_2_, Zr).

The magnitude of anthropogenic enrichment of bottom sediments against the background of surface formations in the vicinity can be used as a complementary or alternative indicator of the extent of contamination of bottom sediments and the geoecological status of aquatic ecosystems. The values of the anthropogenic enrichment index for sediments should be taken into account when planning the economic use of such water bodies and carrying out remediation work.
